# Pharmacotypes across the genomic landscape of pediatric acute lymphoblastic leukemia and impact on treatment response

**DOI:** 10.1038/s41591-022-02112-7

**Published:** 2023-01-05

**Authors:** Shawn H. R. Lee, Wenjian Yang, Yoshihiro Gocho, August John, Lauren Rowland, Brandon Smart, Hannah Williams, Dylan Maxwell, Jeremy Hunt, Wentao Yang, Kristine R. Crews, Kathryn G. Roberts, Sima Jeha, Cheng Cheng, Seth E. Karol, Mary V. Relling, Gary L. Rosner, Hiroto Inaba, Charles G. Mullighan, Ching-Hon Pui, William E. Evans, Jun J. Yang

**Affiliations:** 1 Department of Pharmacy and Pharmaceutical Sciences, St. Jude Children’s Research Hospital, Memphis, TN USA; 2 Khoo Teck Puat–National University Children’s Medical Institute, National University Hospital, National University Health System, Singapore, Singapore; 3 Department of Paediatrics, Yong Loo Lin School of Medicine, National University of Singapore, Singapore, Singapore; 4 Department of Pathology, St. Jude Children’s Research Hospital, Memphis, TN USA; 5 Department of Oncology, St. Jude Children’s Research Hospital, Memphis, TN USA; 6 Department of Biostatistics, St. Jude Children’s Research Hospital, Memphis, TN USA; 7 Quantitative Sciences, Sidney Kimmel Comprehensive Cancer Center at Johns Hopkins, Baltimore, MD USA

**Keywords:** Paediatric cancer, Cancer genomics, Chemotherapy

## Abstract

Contemporary chemotherapy for childhood acute lymphoblastic leukemia (ALL) is risk-adapted based on clinical features, leukemia genomics and minimal residual disease (MRD); however, the pharmacological basis of these prognostic variables remains unclear. Analyzing samples from 805 children with newly diagnosed ALL from three consecutive clinical trials, we determined the ex vivo sensitivity of primary leukemia cells to 18 therapeutic agents across 23 molecular subtypes defined by leukemia genomics. There was wide variability in drug response, with favorable ALL subtypes exhibiting the greatest sensitivity to L-asparaginase and glucocorticoids. Leukemia sensitivity to these two agents was highly associated with MRD although with distinct patterns and only in B cell ALL. We identified six patient clusters based on ALL pharmacotypes, which were associated with event-free survival, even after adjusting for MRD. Pharmacotyping identified a T cell ALL subset with a poor prognosis that was sensitive to targeted agents, pointing to alternative therapeutic strategies. Our study comprehensively described the pharmacological heterogeneity of ALL, highlighting opportunities for further individualizing therapy for this most common childhood cancer.

## Abstract (web summary)

Pharmacotyping analyses of large cohorts of pediatric acute lymphoblastic leukemia identify correlations between drug sensitivities and clinical outcomes across different genomic subtypes.

## Main

Acute lymphoblastic leukemia (ALL), the most common cancer in childhood, comprises a constellation of clinically heterogenous molecular subtypes^1^. The current paradigm of ALL risk stratification integrates clinical features, leukemia somatic genomic aberrations and early treatment response as measured by minimal residual disease (MRD)^2–5^. However, the pharmacological basis of inter-patient variability in MRD is poorly understood and the relationships between somatic genomics and drug resistance phenotypes are unclear. Thus, ALL treatment regimens worldwide almost uniformly use an identical repertoire of conventional chemotherapy drugs with little variation in timing and intensity, and are not influenced by the patient’s sensitivity to specific antileukemic agents. The limited exceptions include the addition of tyrosine kinase inhibitors for ALL with the *BCR*-*ABL1* or *ABL* class fusions^6^. In the current era of personalized medicine, it is imperative to determine whether biologically and pharmacologically informed selection of chemotherapy can further improve ALL treatment outcomes.

MRD reflects the in vivo response to combination chemotherapeutic agents utilized in the ‘induction’ phase in clearing disease to submicroscopic levels, and is widely regarded as the most powerful prognostic factor in ALL treatment^3,7^. However, MRD after combination chemotherapy does not indicate which of the multiple antileukemic agents a patient is responding to, and which are producing little or no therapeutic benefit. De novo and acquired resistance of ALL cells to chemotherapy is known to be a major cause of treatment failure^8^. Directly assessing leukemia cell sensitivity to cytotoxic chemotherapeutic agents ex vivo (prednisolone, vincristine, daunorubicin and L-asparaginase (PVDL)), we and others described the correlation of drug resistance phenotype with treatment response^9–11^ and association with clinical characteristics and somatic genomic features (for example, *BCR*-*ABL1*, *KMT2A* rearrangements)^12–16^. In fact, prospective assessment of these drug sensitivity profiles was shown to have utility in predicting patients at high risk of poor treatment response. However, these studies have several limitations in the context of contemporary ALL treatment because (1) only a narrow selection of conventional chemotherapeutic agents were screened and (2) molecular subtyping was restricted to 4–5 common fusions and aneuploidy^9,17–25^. Therefore, the drug response profiles of most ALL subtypes are unknown.

The current prevailing paradigm of precision oncology is largely driven by cancer genomics, that is, therapy is selected for and matched with a given genomic abnormality^26^. Although this is highly informative for drugs with a clear target (for example, imatinib and *BCR*-*ABL1* leukemias), the mechanisms of drug response are highly complex for most agents. Therefore, assessment of genetic drivers alone may not adequately translate into accurate prediction of therapy effectiveness. With few exceptions, recent trials attempting to select therapy based on molecular target identification have not consistently yielded benefit for patients^27–31^. For this reason, there is a growing interest in functional precision medicine approaches in cancer treatment individualization, such as augmenting genomic testing with direct drug sensitivity profiling using primary tumor specimens^25,32,33^. For example, the recent EXALT trial^34^ used drug profiling to match therapy in patients with aggressive hematological cancers and has shown feasibility for clinical implementation, with promising improvement in patient outcome compared to traditional therapy.

Therefore, we sought to comprehensively characterize the relationship between ex vivo drug sensitivity profiles and in vivo early treatment response across the updated taxonomy of molecular subtypes of ALL, to inform the design of optimal or even new combinatorial therapeutic strategies. To this end, we performed ex vivo pharmacotyping of 18 contemporary chemotherapeutic drugs on primary ALL cells from 805 patients with ALL comprising 23 molecular subtypes and evaluated the impact of leukemia drug sensitivity on initial treatment response as measured by MRD in the context of contemporary ALL treatment regimens.

## Results

### Association of clinical features with ALL drug sensitivity

**Fig. 1:**
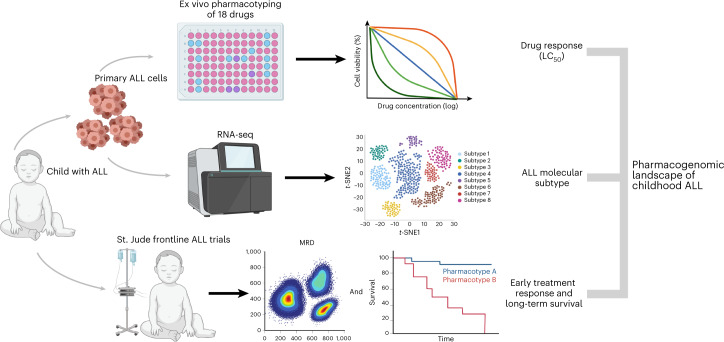
Schematic overview of ALL pharmacotyping, molecular subtyping and evaluation of treatment response. To comprehensively characterize the relationship between drug sensitivity profiles and in vivo treatment response, we performed ex vivo pharmacotyping of 18 drugs on primary ALL cells from 805 patients treated on the St. Jude Total Therapy XV, XVI and XVII trials. Drug profiling was performed via MTT assay or MSC co-culture with flow cytometry, where we evaluated the LC_50_ of each drug (dose required to kill 50% of leukemia cells). We also performed RNA-seq for each patient to determine the molecular subtype. Additionally, as part of each therapeutic trial, every patient had mid-induction (day 15) or post-induction (day 42) MRD determined as a measure of in vivo treatment response to chemotherapy. We then performed integrated analyses of drug sensitivities, somatic genomics, MRD and long-term survival outcomes to characterize the pharmacogenomic landscape of childhood ALL. This figure was created using BioRender.com.

**Extended Data Fig. 1:**
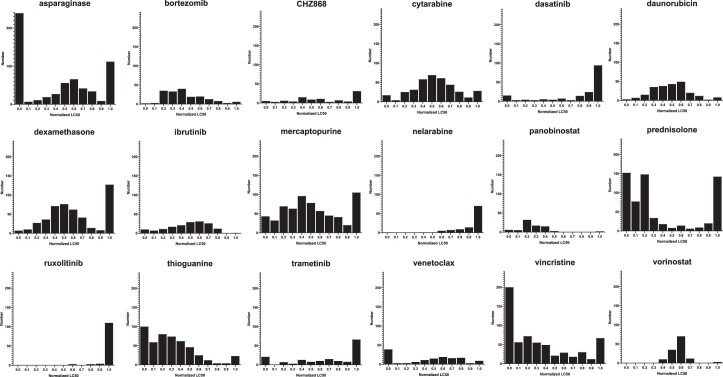
Overall patterns of drug LC50s. Drug sensitivities for each drug were normalized into a range from 0.0 to 1.0 as described in Methods. For each drug, the distribution of frequencies for each interval of 0.1 is plotted in a histogram. Overall, there is a wide range of distribution patterns in this patient cohort. Source data

The sensitivity of leukemia cells from 805 patients was measured for 18 drugs (Fig. 1**and** Table 1), with a total of 5,447 50% lethal concentration (LC_50_) measurements (Supplementary Tables 1 and 2). In parallel, we performed RNA sequencing (RNA-seq) on diagnostic ALL cells to assign each patient to 1 of 23 unique subtypes. Across all drugs, we observed wide interindividual variability in drug sensitivity, with an average coefficient of variation of 55.3% (range = 8.7–105.4%; Extended Data Fig. 1).

**Table 1:** Clinical characteristics of the patient cohort

Characteristic		*n*	%
Sex	Male	440	54.7
	Female	365	45.3
Age group (years)	<1	7	6.7
	1 to <10	603	74.9
	≥10	195	24.2
NCI criteria	Standard risk	465	57.8
	High risk	340	42.2
WBC at diagnosis (×10^9^ /L)	<50	596	74.0
	≥50	209	26.0
Population and ancestry	European	531	66.0
	African	104	12.9
	Admixed American	98	12.2
	Other	65	8.1
	Unknown	7	0.9
Subtype	*ETV6*-*RUNX1*	190	23.6
	Hyperdiploid	178	22.1
	T cell ALL (non-ETP)	113	14.0
	B Other	59	7.3
	*PAX5*alt	36	4.5
	*DUX4*	34	4.2
	*KMT2A*	32	4.0
	*TCF3*-*PBX1*	30	3.7
	*BCR*-*ABL1*	21	2.6
	*CRLF2 (BCR*-*ABL1*-like*)*	21	2.6
	Early T cell precursor (ETP)	16	2.0
	*ETV6*-*RUNX1*-like	14	1.7
	*BCR*-*ABL1*-like (excluding *CRLF2*)	13	1.6
	iAMP21	9	1.1
	*MEF2D*	9	1.1
	Near haploid	8	1.0
	*ZNF384*	8	1.0
	*NUTM1*	5	0.6
	*PAX5* P80R	3	0.4
	Low hypodiploid	2	0.2
	*TCF3*-*HLF*	2	0.2
	*BCL2/MYC*	1	0.1
	*IKZF1* N159Y	1	0.1

We first examined the LC_50_ distribution of each drug by patient characteristics (Supplementary Table 3). Because of the number of drugs evaluated, we also corrected for multiple testing using the Benjamini–Hochberg procedure. We found that patients ≥10 years old had a higher median normalized LC_50_ than those 1 to <10 years of age for L-asparaginase (0.62 versus 0.40, *P* < 0.001) and prednisolone (0.26 versus 0.18, *P* = 0.003). Patients with ≥50 × 10^9^/L leukocyte count at diagnosis had a lower median LC_50_ than those <50 × 10^9^/L for mercaptopurine (0.42 versus 0.50, *P* = 0.004) and dasatinib (0.86 versus 1.0, *P* < 0.001). Assessing by the National Cancer Institute (NCI) risk group for ALL, NCI high-risk patients (age ≥ 10 years or leukocyte count ≥50 × 10^9^/L) had a higher median LC_50_ than NCI standard-risk patients (age < 10 years and leukocyte count <50 × 10^9^/L) for only L-asparaginase (0.56 versus 0.37, *P* < 0.001). There were no differences in drug LC_50_ by sex or ancestry (Supplementary Table 3).

### Drug sensitivity differs across ALL subtypes

**Fig. 2:**
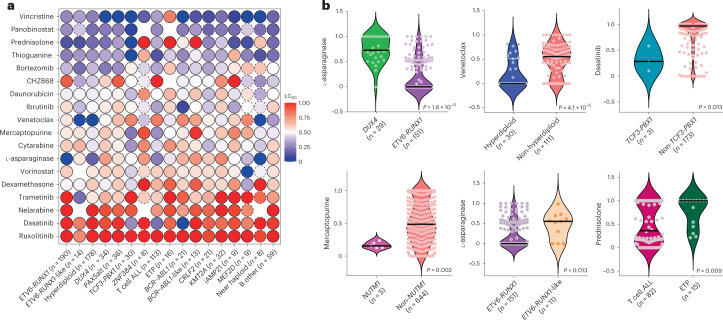
Leukemia drug sensitivities across ALL molecular subtypes. **a**, The median LC_50_ of 18 leukemia drugs are shown for each individual molecular subtype. Low LC_50_ values (that is, higher drug sensitivity) are shown in blue and high LC_50_ values (that is, higher drug resistance) are shown in red. Circles with a dashed line indicate that only a single case with that subtype was tested for that drug. Missing/untested drugs are indicated as empty circles. With the exception of panobinostat, ruxolitinib, bortezomib and daunorubicin, the remaining 14 drugs demonstrated significant inter-subtype variability (*P* < 0.05 nominally and also after Benjamini–Hochberg correction). **b**, Drug LC_50_ distribution is shown in violin plots comparing between selected subtypes. The median LC_50_ for each subtype is shown as a bold black line. The number of patients in each category is indicated in parenthesis and represents biologically independent samples. Nominal *P* values comparing LC_50_ values are as shown and were determined by two-sided Mann–Whitney *U*-test. Source data

**Extended Data Fig. 2:**
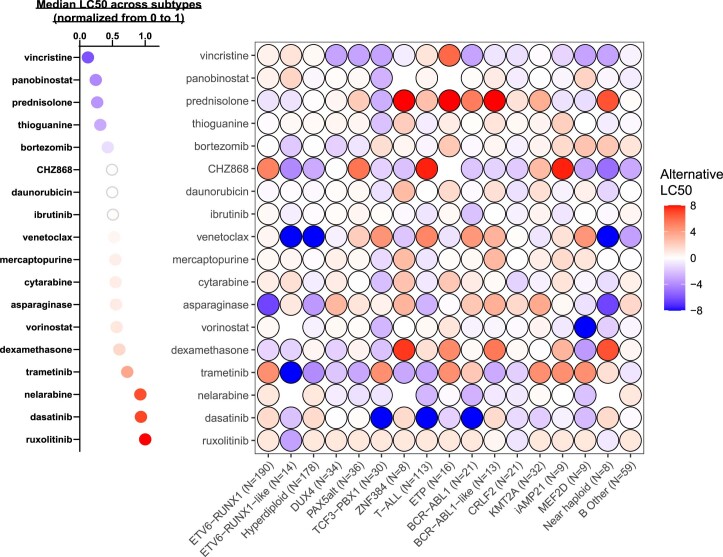
Leukemia drug sensitivities across ALL molecular subtypes by alternative normalization method. To take into account the different concentration ranges measured for different drugs and the difference in their dose-dependent cytotoxicity, an alternative normalization method calculated by log_2_(fold-change from the median) is plotted as shown in the heatmap on the right. The dot plot on the left shows the corresponding median LC50s for each drug as calculated by the original method of normalization (as shown in main Fig. 2a). Source data

We next sought to characterize drug sensitivities across the somatic genomic landscape of ALL. Fourteen of 18 drugs exhibited nominally significant inter-subtype variability (*P* < 0.05), with the exceptions being panobinostat, ruxolitinib, bortezomib and daunorubicin (Fig. 2a, Extended Data Fig. 2 and Supplementary Table 4).

*ETV6*-*RUNX1* and hyperdiploid ALL were highly sensitive to the four drugs commonly used in remission induction (PVDL), recapitulating the known chemotherapy-responsive nature of these subtypes^9,10^. For these two subtypes with known favorable prognosis, the median PVDL LC_50_ was 0.18 for *ETV6*-*RUNX1* and 0.26 for hyperdiploid, both significantly lower than 0.41 for the remaining subtypes (*P* < 0.001 and *P* = 0.004, respectively). By contrast, subtypes known to have poorer prognosis, *BCR*-*ABL1, BCR*-*ABL1*-like and *KMT2A*, exhibited higher PVDL LC_50_ (0.46, *P* = 0.001, 0.66, *P* < 0.001 and 0.54, *P* < 0.001, respectively compared to *ETV6*-*RUNX1*). *DUX4*, a recently discovered subtype with favorable outcomes, had an intermediate PVDL LC_50_ of 0.33 (*P* < 0.001 compared to *ETV6*-*RUNX1*), with a significantly higher L-asparaginase LC_50_ (0.73 versus 0 in *ETV6*-*RUNX1*, *P* < 0.001; Fig. 2b) consistent with its known slow MRD clearance during early remission induction featuring only these 4 drugs^4^.

**Extended Data Fig. 3:**
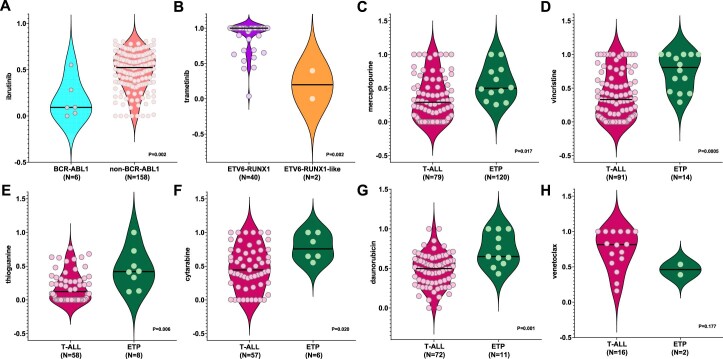
Drug LC50s comparing between ALL subtypes. (**a**) Ibrutinib in *BCR-ABL1* vs non-*BCR-ABL1* (**b**) Trametinib in *ETV6-RUNX1* vs *ETV6-RUNX1*-like (**c**) Mercaptopurine in T-ALL vs ETP-ALL (**d**) Vincristine in T-ALL vs ETP-ALL (**e**) Thioguanine in T-ALL vs ETP-ALL (**f**) Cytarabine in T-ALL vs ETP-ALL (**g**) Daunorubicin in T-ALL vs ETP-ALL (**h**) Venetoclax in T-ALL vs ETP-ALL Drug LC50s are plotted in a violin plot for selected subtypes. The median LC50 for each subtype is shown as a bold black line. The number of patients in each category is indicated in parenthesis and represents biologically independent samples. Nominal P values comparing LC50s are as shown and determined by the 2-sided Mann-Whitney test. Source data

We also observed highly subtype-dependent patterns of sensitivities to targeted agents. Hyperdiploidy was significantly more sensitive (*P* < 0.001) to venetoclax than non-hyperdiploidy (Fig. 2b). High sensitivities to dasatinib were seen for T cell ALL (LC_50_ 0.20, *P* < 0.001 compared to non-T cell ALL), *TCF3*-*PBX1* (LC_50_ 0.28, *P* = 0.013 compared to non-*TCF3*-*PBX1*; Fig. 2b) and *BCR*-*ABL1* (LC_50_ 0, *P* < 0.001 compared to non-*BCR*-*ABL1*), consistent with previous findings by us and others^35,36^. *BCR*-*ABL1* was also sensitive to ibrutinib (LC_50_ 0.09, *P* = 0.002 compared to non-*BCR*-*ABL1*; Extended Data Fig. 3a). *NUTM1* had low LC_50_ for several antimetabolites: LC_50_ 0.09 for cytarabine (*P* = 0.007 compared to non-*NUTM1* subtypes), LC_50_ 0.16 for mercaptopurine (*P* = 0.002; Fig. 2b) and LC_50_ 0 for thioguanine (*P* = 0.0003).

**Extended Data Fig. 4:**
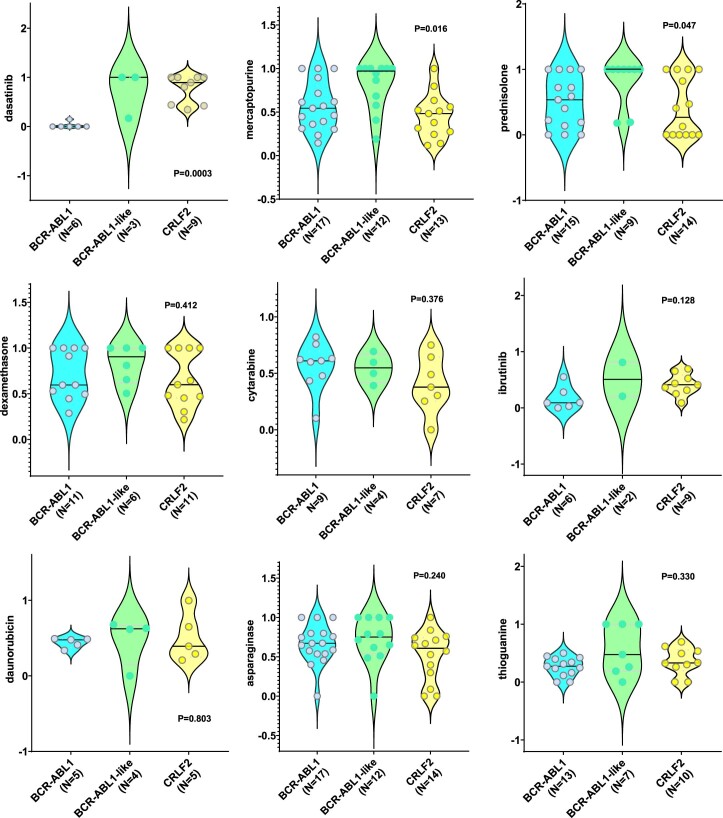
Drug LC50s comparing BCR-ABL1, BCR-ABL1-like, and CRLF2-r ALL. Drug LC50s are plotted in violin plots for these three subtypes. The median LC50 for each subtype is shown as a bold black line. The number of patients in each category is indicated in parenthesis and represents biologically independent samples. Amongst the 3 BCR-ABL1-like cases tested for dasatinib, 2 harbored ABL class fusions (one with PDGFRB, and one with CSFR1). Nominal P-values are as shown and determined by 2-sided Kruskal-Wallis test. Source data

Although characterized by nearly identical global gene expression profiles, *ETV6*-*RUNX1*-like and *ETV6*-*RUNX1* ALL showed notable differences in drug sensitivity: *ETV6*-*RUNX1*-like ALL was more resistant to L-asparaginase (LC_50_ 0.55 compared to 0 in *ETV6*-*RUNX1*, *P* = 0.013; Fig. 2b) but had increased sensitivity to trametinib (LC_50_ 0.20 versus 1.0, *P* = 0.002; Extended Data Fig. 3b). *BCR*-*ABL1*, *BCR*-*ABL1*-like and *CRLF2* are also related to each other based on global transcriptional profile and somatic genomic features, yet these 3 subtypes had varying sensitivities to dasatinib (LC_50_ 0 versus 1.0 versus 0.89, respectively, *P* < 0.001), mercaptopurine (LC_50_ 0.54 versus 0.97 versus 0.48, *P* = 0.016) and prednisolone (LC_50_ 0.54 versus 1.0 versus 0.27, *P* = 0.047) but with similar response to other cytotoxic and targeted drugs (Extended Data Fig. 4). Comparing with other T cell ALL (Extended Data Fig. 3c–h**)**, early T cell precursor (ETP) ALL was more resistant to antimetabolite and non-antimetabolite cytotoxic drugs: vincristine (LC_50_ 0.81 versus 0.33, *P* < 0.001), prednisolone (LC_50_ 1.0 versus 0.36, *P* = 0.009; Fig. 2b), thiopurines (mercaptopurine LC_50_ 0.50 versus 0.29; *P* = 0.017 and thioguanine LC_50_ 0.42 versus 0.12; *P* = 0.006), cytarabine (LC_50_ 0.76 versus 0.45, *P* = 0.023) and daunorubicin (LC_50_ 0.65 versus 0.50, *P* = 0.001). ETP showed a trend for higher venetoclax sensitivity compared to T cell ALL, although this was not statistically significant in this relatively small cohort (LC_50_ 0.46 versus 0.81). Taken together, our data point to new subtype-specific therapeutic opportunities in ALL. To explore the effects of multiple testing, we also performed Benjamini–Hochberg correction for these subtype-related comparisons (Supplementary Table 5) and the majority of the associations remained significant.

### Association of ALL ex vivo drug sensitivity with MRD

We evaluated the association of drug LC_50_ with in vivo response, as reflected by MRD during remission induction therapy. For each patient, MRD was measured at day 15 and day 42. In this study, we evaluated the coefficients of linear regression (*β*) for each drug, where each unit change in coefficient represents a unit change of MRD (log_10_-transformed),that is, a coefficient of 0.30 (or log_10_(2)) indicates that the MRD is twice as high in resistant patients compared to sensitive patients; a coefficient of −0.30 (or log_10_(0.5)) indicates that the MRD is half as high. A coefficient of zero indicates that there is no difference in MRD between resistant and sensitive patients.

**Fig. 3:**
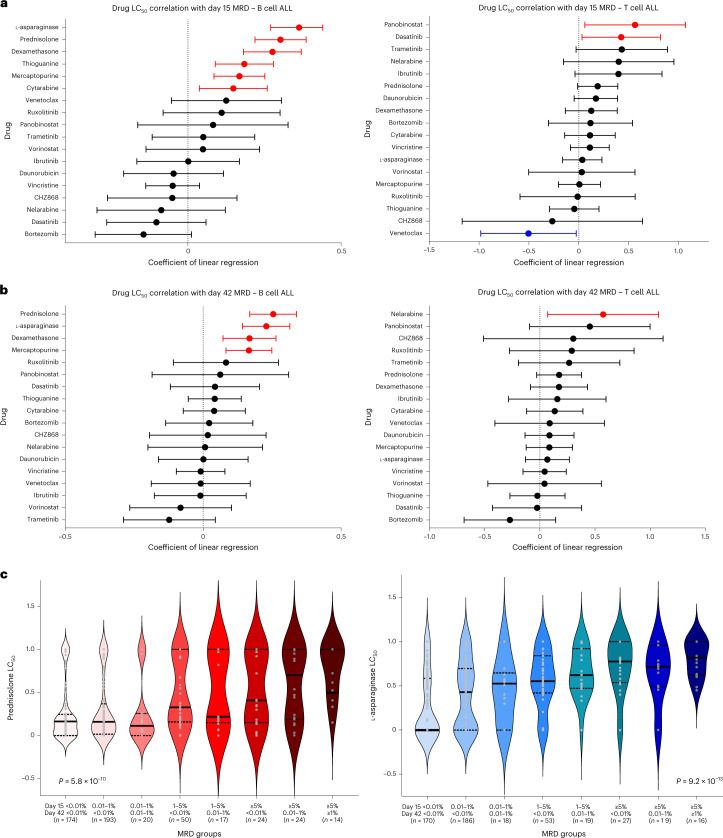
Correlation of ALL drug sensitivities with MRD during induction therapy. **a**,**b**, Forest plots depict drug LC_50_ correlation with day 15 MRD (B cell ALL, *n* = 671 patients; T cell ALL, *n* = 105 patients, representing biologically independent samples) (**a**) and day 42 MRD (B cell ALL, *n* = 669; T cell ALL, *n* = 105, representing biologically independent samples) (**b**). Left, Correlations with B cell ALL. Right, Correlations with T cell ALL. The coefficient of linear regression between each drug and MRD at each time point is shown as solid dots, with the 95% CIs indicated by the horizontal bars. Each unit change in coefficient represents a unit change of MRD (log_10_-transformed), that is, a coefficient of 1.0 represents a tenfold increase in MRD. Significant positive correlations are shown in red, negative correlations are shown in blue and those not reaching statistical significance are shown in black. **c**, Association of longitudinal MRD with B cell ALL sensitivity to prednisolone and L-asparaginase. LC_50_ of prednisolone (left, shades of pink/red) and L-asparaginase (right, shades of blue/teal) are plotted for 8 groups with different combinations of day 15 and day 42 MRD. The median LC_50_ of each group is shown as a bold horizontal black line for each violin plot, with the number of patients (biologically independent samples) in each category shown in parenthesis. In this study, the LC_50_ of both drugs increase progressively across MRD groups with rising MRD levels (*P* = 5.8 × 10^−10^ for prednisolone and *P* = 9.2 × 10^−13^ for L-asparaginase, determined by two-sided Kruskal–Wallis test). Additionally, the pattern of influence appears to differ between both drugs. Prednisolone LC_50_ was strikingly higher in MRD groups comprising high levels of MRD positivity but was relatively equal for MRD groups with low or no MRD. By contrast, L-asparaginase LC_50_ was strikingly lower only at complete day 15 and day 42 MRD negativity but was relatively equal for MRD groups with any degree of MRD positivity. Source data

Drugs that were significantly related to MRD differed markedly between B cell ALL and T cell ALL (Fig. 3a,b). In B cell ALL, drugs that positively correlated with both day 15 and day 42 MRD included L-asparaginase (*β* = 0.30; *P* < 0.001 for day 15 and *β* = 0.23; *P* < 0.001 for day 42), prednisolone (*β* = 0.30; *P* < 0.001 for day 15 and *β* = 0.25; *P* < 0.001 for day 42), dexamethasone (*β* = 0.28; *P* < 0.001 for day 15 and β = 0.17; *P* < 0.001 for day 42) and mercaptopurine (*β* = 0.17; *P* < 0.001 for day 15 and *β* = 0.17; *P* < 0.001 for day 42). Additionally, thioguanine (*β* = 0.18; *P* < 0.001) and cytarabine (*β* = 0.15; *P* = 0.009) were also associated with day 15 MRD. For T cell ALL, panobinostat (*β* = 0.56; *P* = 0.028) and dasatinib (*β* = 0.42; *P* = 0.034) were positively correlated with day 15 MRD and venetoclax negatively with day 15 MRD (*β* = −0.50; *P* = 0.039). Nelarabine was positively correlated with day 42 MRD (*β* = 0.57; *P* = 0.025).

**Extended Data Fig. 5:**
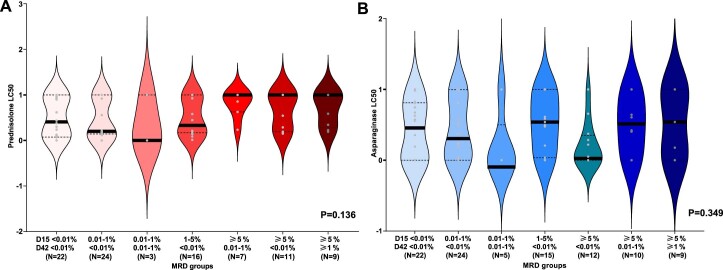
Association of longitudinal minimal residual disease with T-ALL sensitivity to prednisolone and asparaginase. LC50s of each drug are plotted in the violin plots for each combination of D15 and D42 MRD categories. Median LC50s of each group is shown as a bold horizontal black line for each violin plot, with the number of patients (biologically independent samples) in each category indicated. Prednisolone is shown in panel A in shades of pink/red, while asparaginase is shown in panel B in shades of blue. Unlike B-ALL, prednisolone and asparaginase are not correlated with MRD. P-values are nominal and determined by the 2-sided Kruskal-Wallis test. Source data

Leveraging longitudinal MRD data (days 15 and 42), we also classified patients into groups with increasing resistance to induction therapy, that is, those who cleared leukemia early and remained MRD negative were the most sensitive whereas those with high MRD at both time points were the most resistant. Prednisolone and L-asparaginase LC_50_ progressively increased with rising MRD category in B cell ALL (*P* < 0.001 for both; Fig. 3c) but not in T cell ALL (*P* = 0.136 for prednisolone and *P* = 0.349 for L-asparaginase; Extended Data Fig. 5), indicating lineage-specific effects of drug resistance on MRD. More importantly, prednisolone resistance of leukemia cells ex vivo most strongly predicted MRD persistence (that is, MRD positivity at day 42). By contrast, leukemia cell sensitivity to L-asparaginase was most significantly associated with early MRD clearance (that is, MRD negativity at day 15).

### Pharmacotypes define distinct ALL subsets and prognosis

**Fig. 4:**
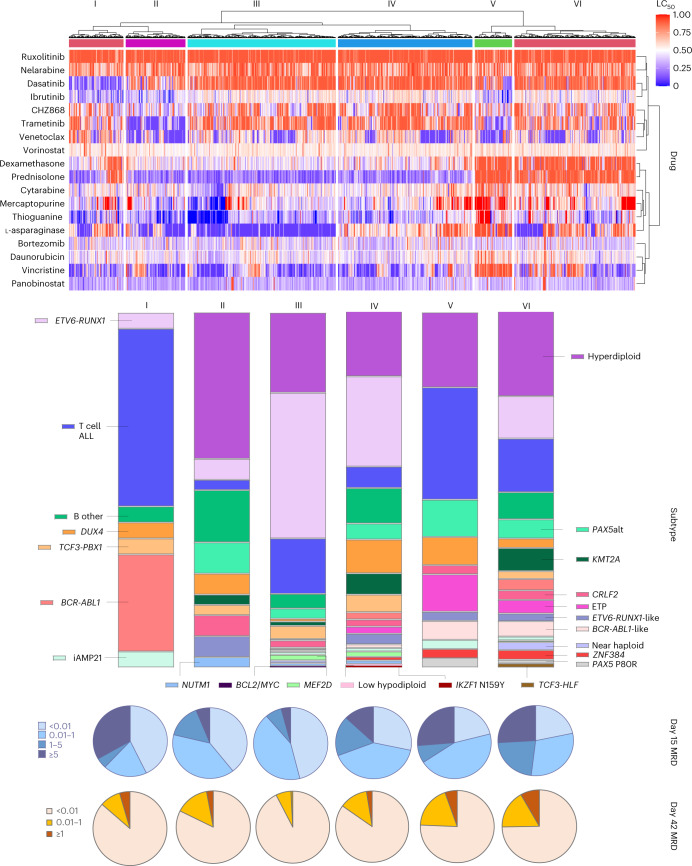
Drug sensitivity profile defines distinct ALL patient clusters. Hierarchical clustering revealed six taxonomic groups with distinct patterns of drug sensitivity, as shown on the heatmap. Hierarchical clustering of patients was performed based on all imputed LC_50_ values using Manhattan distance measure. Each patient received a cluster assignment for each round of imputation. Patients were assigned to a final cluster if that cluster assignment appeared in the same cluster for at least five of ten rounds of imputation. Each vertical block of the heatmap corresponds to a cluster, numbered I to VI. The heatmap depicts higher drug sensitivity in blue and higher drug resistance in red. The distribution of subtypes within each cluster is shown as bar graphs. MRD at days 15 and 42 of each cluster are indicated by the pie charts below the heatmap. Source data

To address missingness in drug sensitivity measurement, we imputed LC_50_ values using sequential regression multiple imputation (n = 10). Applying unsupervised hierarchical clustering analysis to a matrix of 8,050 × 18 data points, we observed 6 clusters of ALL cases with unique patterns of drug response phenotypes (Fig. 4).

Cluster I was defined by prominent sensitivities to dasatinib and ibrutinib, while cluster II showed sensitivity to venetoclax and trametinib. Cases within cluster III were universally sensitive to L-asparaginase and prednisolone. Cluster IV exhibited similar sensitivity to prednisolone but differed from cluster III based on L-asparaginase resistance. Cluster V was the most resistant group, with the highest LC_50_ across all cytotoxic drugs (steroid, L-asparaginase, vincristine, daunorubicin and thiopurines). However, this group also showed some sensitivity to targeted drugs such as trametinib, venetoclax and ibrutinib. Cluster VI had a more heterogeneous drug sensitivity profile, with consistent resistance to glucocorticoids but mixed response to other cytotoxic or targeted agents.

Overall, there was high heterogeneity of somatic genomics represented by each drug sensitivity-based cluster (*P* < 0.001). Cluster I was defined mostly by subtypes with known sensitivity to dasatinib, that is, *BCR*-*ABL1* (27%) and T cell ALL (50%). Cluster II had a high proportion of hyperdiploidy (41%). Cluster III had the highest proportion of *ETV6*-*RUNX1* (41%), followed by hyperdiploid ALL (23%), likely consisting of low-risk ALLs that are chemotherapy-sensitive. Cluster IV was highly heterogenous, representing 19 different subtypes. In this cluster, there was a preponderance of *DUX4* (9%) and *KMT2A* (6%). Cluster V had a greater proportion of T cell ALL (31.6%), ETP ALL (10.5%) and *BCR*-*ABL1*-like (5.3%). Cluster VI also exhibited diverse subtype composition, with a high prevalence of *KMT2A* (6.5%) and *BCR*-*ABL1*-like (4.3%), and *TCF3*-*HLF* exclusively appeared in this cluster, all of which are linked to poor prognosis.

MRD response also differed significantly across drug sensitivity clusters (*P* < 0.001 for both day 15 and day 42 MRD). Clusters I, II and III represented patients with rapid early leukemia clearance (proportion of day 15 MRD negativity at 43%, 39% and 46%, respectively) compared to clusters IV, V and VI (28%, 21% and 22%, respectively), although cluster I also had a strikingly large proportion of treatment-resistant patients (33% with MRD day 15 ≥ 5%). At day 42, clusters I, II and III continued to show high MRD negativity (86%, 82% and 93%, respectively). Cluster IV patients had high MRD positivity at day 15 but responded to subsequent induction therapy with a much-improved MRD clearance at day 42 (MRD-negative proportion = 85%), a characteristic response we have previously reported for the *DUX4* subtype^4^. By contrast, clusters V and VI exhibited persistent poor response with a higher proportion of cases showing day 42 MRD positivity.

**Fig. 5:**
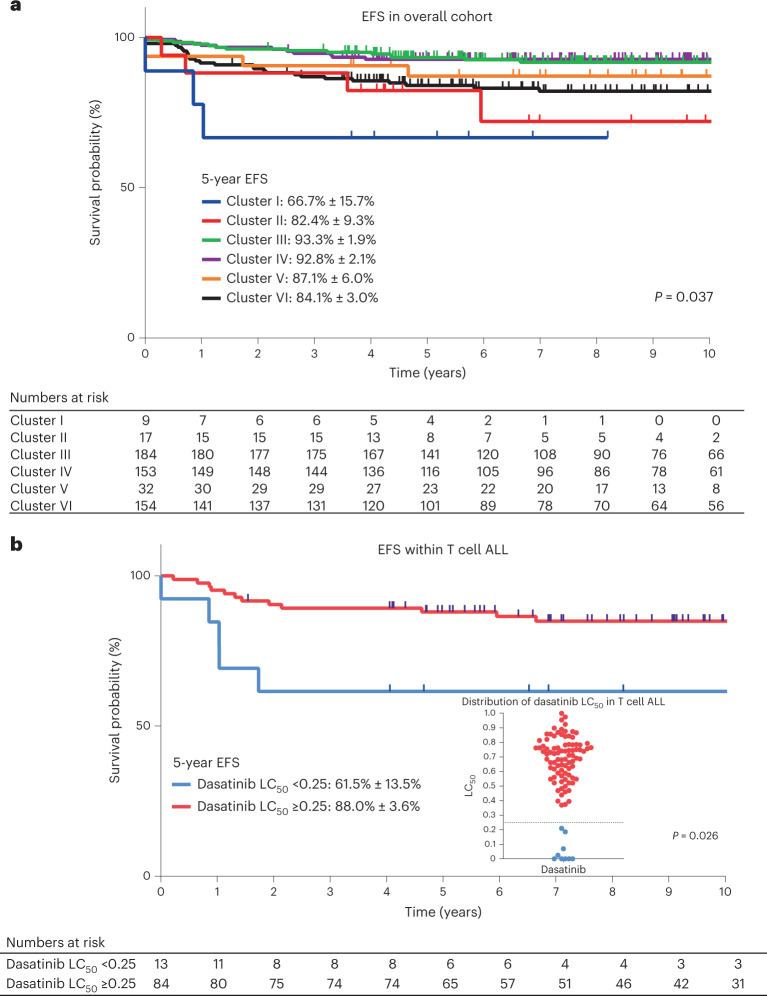
Association of drug sensitivity profiles with EFS. **a**, EFS across 6 drug sensitivity clusters in the entire cohort (*n* = 549) (**a**) and EFS across 2 dasatinib sensitivity groups in T cell ALL (*n* = 97) (**b**). Kaplan–Meier curves were plotted for each drug sensitivity group and the 5-year EFS with s.e. are shown in the figure. The numbers of patients at risk are shown beneath each graph. Each cluster is represented by a different color. **b**, The dasatinib-resistant group (LC_50_ ≥ 0.25) is indicated in red and the dasatinib-sensitive group (LC_50_ < 0.25) is indicated in blue. The dot plot in **b** demonstrates the distribution of dasatinib LC_50_ in patients with T cell ALL, with the horizontal dotted line shown at 0.25. *P* values were determined by two-sided Cox proportional-hazards regression test and adjusted for treatment arm.

Finally, we evaluated the association of drug sensitivity clustering pattern with ALL treatment outcomes, focusing on the Total Therapy XV and XVI trials for whom long-term survival data are mature^37,38^. Other than a higher proportion of patients with presenting white blood cell (WBC) count <50 × 10^9^/L, there were no differences in clinical characteristics between patients included and excluded in this analysis (Supplementary Table 6). Event-free survival (EFS) differed significantly across the 6 drug sensitivity clusters (*P* = 0.037; Fig. 5a) and the drug sensitivity cluster remained prognostic even after adjusting for day 42 MRD (Supplementary Table 7). Clusters III and IV, which were characterized by steroid and thiopurine sensitivities, had the best survival outcomes in keeping with rapid MRD clearance. By contrast, cluster I (defined mainly by dasatinib sensitivity) had the poorest outcomes in this cohort. In fact, three of three events in this group occurred in T cell ALL cases with dasatinib sensitivity, with none in *BCR*-*ABL1* ALL. We therefore evaluated the prognostic significance of dasatinib sensitivity in the entire cohort of 97 patients with T cell ALL, none of whom received treatment with dasatinib, where we found that higher dasatinib sensitivity (LC_50_ < 0.25) was associated with an inferior EFS (hazard ratio = 3.3, 95% confidence interval (CI) = 1.3–10.8, *P* = 0.026; Fig. 5b). The prognostic impact of dasatinib sensitivity in T cell ALL was greater than that of MRD and remained significant after adjusting for MRD (Supplementary Table 8).

Taken together, these data revealed intrinsic relationships in ALL sensitivity to different therapeutic agents and pointed to the pharmacological basis of inter-subtype variability in patients with ALL response to induction therapy and long-term survival outcomes.

## Discussion

Although the overall cure rate of childhood ALL has surpassed 90%, more children die of ALL than most solid tumors. Therefore, further improvements in treatment are needed. Given the marked heterogeneity of somatic genomic profiles of childhood ALL and the incomplete knowledge of their relationships with leukemia drug sensitivity, genomics-centric approaches may have limited utility for precision medicine of ALL. Therefore, defining a patient’s drug sensitivity phenotype, that is, pharmacotyping, may be an additional useful strategy for treatment individualization^39^. In this study, we present an integrated analysis of patient primary ALL drug sensitivity profiles, leukemia genomic features and in vivo treatment response, revealing relationships between ALL genomics and leukemia drug response in vitro and in vivo, thereby providing new insights into the pharmacological basis of inter-patient variability in ALL treatment outcomes.

**Extended Data Fig. 6:**
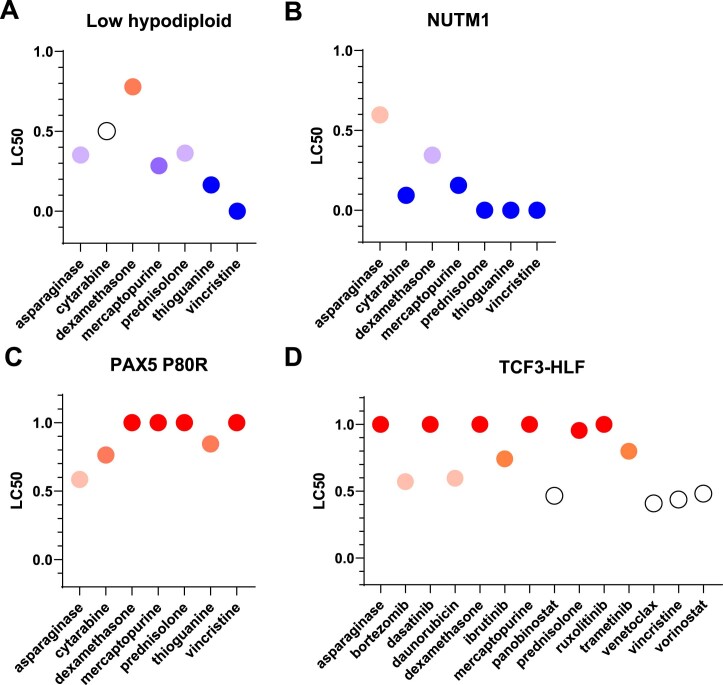
Drug LC50s of rare subtypes. (**a**) Low hypodiploid (**b**) *NUTM1* (**c**) *PAX5* P80R (**d**) *TCF3-HLF* LC50 of these four subtypes are plotted for drugs tested. Higher sensitivity is indicated in blue and higher resistance is indicated in red. Source data

Our pharmacotyping results may enable more precise refinement of ALL treatment. For example, patients with high MRD during induction are often given extra L-asparaginase without consideration of their leukemia drug sensitivity pattern^40^. While this approach has been shown to reduce end-of-induction MRD levels in most leukemia subtypes^38^, adding more L-asparaginase for all patients may not be optimal, given that the *KMT2A* subtype is generally resistant, in which case adding L-asparaginase may increase toxicity without providing therapeutic benefit. Similarly, *DUX4* ALL is resistant to L-asparaginase and associated with poor day 15 MRD, yet demonstrates excellent MRD clearance at day 42. This is plausibly attributable to its sensitivity to antimetabolites and cyclophosphamide used in the second half of St. Jude’s induction therapy, although this requires experimental validation. Other possible factors contributing to long-term remission beyond drug sensitivity include host immunity against residual leukemia^41^, which may be more activated in certain subtypes like *DUX4* because of greater immunogenicity of the neoantigens in these leukemia cells. Notably, *ZNF384*, a recently discovered intermediate risk subtype, displays resistance to glucocorticoids but sensitivity to venetoclax and bortezomib. Similarly, *TCF3*-*HLF* and *PAX5* P80R ALLs were generally drug-resistant (LC_50_ > 0.70 for most agents; Extended Data Fig. 6), consistent with their poor prognosis^6^. However, *TCF3*-*HLF* ALL showed sensitivity to venetoclax as described previously^42^. These individual subtype drug sensitivity profiles, along with the distinct functional drug clustering patterns, may prove useful in designing new subtype-specific treatment protocols. Although the efficacy of venetoclax in T cell ALL has already been explored (especially in ETP^43^), our results show that venetoclax LC_50_ was negatively correlated with day 15 MRD in T cell ALL, further supporting the possibility of using this targeted drug to augment treatment for those with poor initial response to conventional induction chemotherapy^44^. Because of potential efficacy along with reduced toxicity profiles compared to conventional cytotoxics, targeted drugs are increasingly being investigated in both frontline and relapsed trials^45–47^, some with early promising results. However, for most patients it is unclear who will benefit from these therapies. Pharmacotyping studies like ours may provide a blueprint for individualizing therapy by informing the selection of patients who are anticipated to respond to these drugs. Even though our study revealed remarkable heterogeneity in ALL drug response (particularly across molecular subtypes), the exact algorithm for using pharmacotyping results to direct leukemia therapy for individual patients is yet to be developed. Early data from the EXALT trial^34^ pointed to the feasibility of this approach and similar studies are much needed for ALL. Another caveat to note is that a higher LC_50_ of a particular drug does not necessarily mean it is less active than a different drug with a lower LC_50_ because LC_50_ is normalized within each drug and direct LC_50_ comparison across drugs is not meaningful.

Of all the drugs used in ALL therapy, L-asparaginase and prednisolone had the strongest impact on MRD, but with differential influence. For prednisolone, patients with high sensitivity ex vivo (Fig. 3c, left, first four columns) exhibited a range of day 15 MRD but almost always cleared leukemia by day 42, suggesting that (1) glucocorticoids may be slow acting in some cases and (2) continuing exposure may be needed for most patients to eventually achieve MRD-negative remission. For L-asparaginase, patients with the greatest sensitivity (Fig. 3c, right, left-most column) cleared leukemia rapidly with negative day 15 MRD, suggesting that the initial doses of L-asparaginase (given at the beginning of induction therapy) were highly effective in reducing leukemia burden in this group. Also, we found that drugs such as thiopurines predicted response to early induction therapy at day 15 even though they have not yet been administered at that time point. This likely reflects inherent features of leukemia cells that drive response to other antileukemic agents used in induction therapy. For example, thiopurine LC_50_ was significantly correlated with that of L-asparaginase (used in the first week), pointing to overlap in the biological mechanism of their cytotoxic effects. Additionally, it should be noted that Total Therapy trials have a longer duration of induction therapy compared to many non-St. Jude ALL protocols; therefore, end-of-induction MRD in our dataset would reflect response to more intense chemotherapy. That said, the prognostic impact of MRD is universal across a variety of ALL treatment regimens, regardless of the precise time point it is measured^3,6^. Also, therapy in the first two weeks of the induction phase is highly consistent across frontline ALL protocols from different cooperative trial groups. Therefore, we reason that our observed associations of drug sensitivity with MRD are broadly relevant and especially so with day 15 MRD. One limitation to note, however, is the single-agent nature of our drug sensitivity profiling, which may not reflect the impact of synergistic effects of some drug combinations. Second, some important drugs, such as methotrexate, were not studied due to their unreliable cytotoxicity in ex vivo drug assays. Therefore, for such drugs, alternative in vivo studies may represent the only suitable approach for comparing resistance across patient cohorts^48^.

In our cohort, drug sensitivity clusters were strongly associated with survival outcomes, even after accounting for MRD, highlighting their potential prognostic utility in ALL risk stratification. Of particular interest is the association of dasatinib sensitivity with poorer survival in T cell ALL for a number of reasons: (1) ALL pharmacotypes identified a group of patients at higher risk of relapse, whose poor prognosis was not predicted by usual markers such as MRD; (2) leukemia sensitivity to dasatinib in this T cell ALL subset revealed a therapeutic vulnerability that could be leveraged to improve survival given their inferior treatment response to conventional cytotoxic chemotherapy that did not include dasatinib. These findings should be explored prospectively on a large scale in clinical trials.

In summary, we comprehensively characterized drug response profiles across molecular subtypes in childhood ALL, evaluated the association of drug sensitivities with early treatment response in patients, and explored pharmacotyping-based subgrouping of ALL and its impact on survival outcomes. Our results have potential clinical relevance because they provide new insights for the design of novel combination therapy, particularly in conjunction with ALL molecular subtyping. With these approaches, future therapy can be individualized to further improve outcome for every child with ALL.

## Methods

### Patient and clinical treatment protocols

The ALL cases included for pharmacotyping consisted of 805 children and adolescents from St. Jude Children’s Research Hospital, treated on 3 consecutive ALL Total Therapy protocols: Total XV^37^ (ClinicalTrials.gov NCT00137111), XVI^38^ (NCT00549848) and XVII (NCT03117751). This study was approved by the institutional review board at St. Jude Children’s Research Hospital. Written informed consent was obtained from parents, guardians and/or patients, as appropriate.

### Genomic profiling, MRD and determination of genetic ancestry

Leukemia blasts were obtained from either bone marrow or peripheral blood after Ficoll gradient centrifugation. Samples were subjected to further enrichment by magnetic-activated cell sorting if blast percentage was <85% (CD19 for B cell ALL and CD7 for T cell ALL, respectively). Leukemia cells were subjected to drug sensitivity profiling ex vivo for a panel of up to 18 antileukemic agents (see the Pharmacotyping section). MRD levels were determined by flow cytometry in bone marrow samples in the middle of induction (day 15 MRD) on days 15 (Total XVI and XVII) or 19 (Total XV), and end of remission induction (day 42 MRD) on days 42–46 (ref. ^4^). A negative MRD was defined as a level of less than 1 leukemia cell among 10,000 mononuclear cells (<0.01%). MRD was not a prespecified endpoint of the Total Therapy XVII trial.

For genomic profiling, nucleic acid was extracted from bone marrow or peripheral blood ALL cells at diagnosis (as the leukemia sample) or normal leukocytes during clinical remission (as the germline sample). Genetic ancestry (European, African, Native American, Asian) was estimated with iAdmix^49^ by comparing allele frequencies of germline single-nucleotide polymorphisms with reference populations from the 1000 Genomes Project^50^. Patients were then classified into mutually exclusive populations based on genetic ancestry composition and defined as: European (European >90%); African (African >70%); Admixed American (Native American >10% and Native American greater than African). The rest were defined as ‘Other’^51,52^.

### Pharmacotyping of primary ALL cells

In total, drug response of primary patient ALL cells were evaluated for 18 drugs representing 5 classes: (1) antimetabolites (cytarabine, mercaptopurine, nelarabine and thioguanine); (2) non-antimetabolite cytotoxics (daunorubicin, dexamethasone, prednisolone, vincristine, L-asparaginase and bortezomib); (3) tyrosine kinase inhibitors (CHZ868, dasatinib, ibrutinib, ruxolitinib and trametinib); (4) histone deacetylase inhibitors (panobinostat and vorinostat); and (5) BH3-mimetics (venetoclax).

For all drugs, 6 drug concentrations were tested (Supplementary Table 9): L-asparaginase (0.032–10 IU ml^−1^); bortezomib (0.98–1,000 nM); CHZ868 (0.1–10,000 nM); cytarabine (0.04–41.1 µM); dasatinib (0.1–10,000 nM); daunorubicin (0.004–3.55 µM**)**; dexamethasone (0.00035–11.6 µM); ibrutinib (1.5625–50 µM); mercaptopurine (91.8–2938 µM); nelarabine (1.03–250 µM); panobinostat (0.98–1,000 nM); prednisone (91.8–2938 µM); ruxolitinib (0.1–10,000 nM); thioguanine (9.35–299 µM); trametinib (0.01–1,000 nM); venetoclax (0.001–100 nM); vincristine (0.0017–54.169 µM); and vorinostat (102.88–25,000 nM).

For L-asparaginase, bortezomib, cytarabine, daunorubicin, dexamethasone, mercaptopurine, nelarabine, panobinostat, prednisolone, thioguanine, vincristine and vorinostat, sensitivities of primary ALL cells to these drugs were determined with the use of the 4-day in vitro 3-(4,5-dimethylthiazol-2-yl)-2,5-diphenyl tetrazolium bromide (MTT) drug resistance assay^9,11^. Briefly, after 4 d of culture at 37 °C in humidified air containing 5% CO_2_, 0.45 mg ml^−1^ MTT was added. After an additional 6 h, formazan crystals (produced by viable cells only) were dissolved in acidified isopropanol and quantified by spectrophotometry. Samples with more than 70% leukemic cells in the control wells and an optical density >0.050 absorbance units (adjusted for blank values) were used to calculate the LC_50_ of cells by a 4-parameter dose–response model.

The remainder of drugs were profiled using a mesenchymal stem cell (MSC) co-culture system assay with flow cytometry, as described previously^35^. Briefly, hTERT-immortalized MSCs were first seeded in a 96-well plate at a density of 10,000 cells per well in 100 μl complete medium (RPMI-1640, L-glutamine, 10% FCS and 1 μM hydrocortisone). After 24 h, leukemia cells were added at 160,000 cells per well to the stromal cell layer in 80 μl AIM V medium along with 20 μl of drug solution prepared in the same medium. After 96 h of incubation at 37 °C with 5% CO_2_, cells were collected and stained with CD19 or CD7 to identify leukemia blasts (for B cell and T cell ALL, respectively). The total number of live leukemia cells were evaluated using flow cytometry after annexin V and DAPI staining (antibodies used: human CD7-PE (clone 4H9, catalog no. 395604; BioLegend), human CD19-PE (clone SJ25C1, catalog no. 363004; BioLegend) and annexin V APC (research resource identifier AB_2868885, catalog no. 550475; BD Biosciences). FlowJo was used for analysis. Drug-induced death was estimated by comparing leukemia cells treated with vehicle alone. LC_50_ was determined the same as in the MTT assays. Quality control was performed to remove cases with low viability (<1,000 viable blast cells in each well in the absence of drugs on day 4).

LC_50_ values were used to compare the sensitivity of ALL cells across the entire population, within specific ALL subtypes and with early treatment response (as assessed by MRD). For cases where even the lowest drug concentration killed >50% of leukemia cells, LC_50_ was assigned as half of the minimum tested concentration. Conversely, for cases with >50% viability even at the highest drug concentration, LC_50_ was assigned as twice of the highest tested concentration. These raw LC_50_ values were then log-transformed and normalized into a range between 0 and 1.0 for statistical analyses (see the Statistical analyses section).

### RNA-seq and ALL subtype analysis

The TruSeq stranded mRNA or total RNA library prep kit (Illumina) was used for whole-transcriptome library preparation of RNA extracted from diagnostic blasts. Paired-end sequencing was performed using the Illumina HiSeq 2000/2500 platform with a 2 × 101 base pair (bp) read length or NextSeq 500 with a 2 × 151 bp read length. Sequencing reads were mapped to the GRCh37 human genome reference by STAR (v.2.4.2a), through the suggested two-pass mapping pipeline. Samples were excluded if the percentage of exonic regions with 10× or more coverage was <30%, read duplication rate >55% or a strong 3′-bias of transcript coverage. Gene annotation downloaded from the Ensembl website was used for STAR mapping and the following read count evaluation. CICERO^53^ and FusionCatcher were used to detect fusions and all the reported rearrangements were manually curated using BLAT and the Integrative Genomics Viewer. Gene expression was quantified as fragments per kilobase of transcript per million mapped reads (FPKM) using RSEM (v.1.2.2887). Gene level FPKM values were used for downstream analyses, after removing lowly expressed genes and genes with invariable expression.

We determined 23 ALL subtypes using RNA-seq data for *n* = 741 patients, as described previously^1,52^. B cell ALL cases were divided into distinct subtypes by rearrangements (*BCR*-*ABL1*, *ETV6*-*RUNX1*, *KMT2A*, *TCF3*-*PBX1*, *DUX4*, *ZNF384*, *MEF2D*, intrachromosomal amplification of chromosome 21 (iAMP21), *BCL2/MYC*, *NUTM1*, *CRLF2*, *HLF*), aneuploidy (hyperdiploid (chromosome number ≥51), low hypodiploid (chromosome number 31–39) and near haploid (chromosome number 24–30)), gene expression profiles (*ETV6*-*RUNX1*-like, *PAX5*alt and *BCR*-*ABL1*-like) and sequence mutations (*PAX5* P80R and *IKZF1* N159Y). Cases with the *BCR-ABL*-like gene expression signature and also a *CRLF2* rearrangement were classified as *CRLF2*. The remainder of unassigned B cell ALL subtypes were labeled as B Other. ETP ALL was determined by RNA-seq and immunophenotyping as described previously^54^. For the 64 patients without RNA-seq available, we assigned subtypes by fluorescence in-situ hybridization and karyotyping for traditional fusions (*ETV6*-*RUNX1*, *KMT2A*, *TCF3*-*PBX1*, *BCR*-*ABL1*) and/or gene expression profile from an Affymetrix HG-U133A array^52^.

### Statistical analyses

MRD levels were log-transformed and those with less than the detection limit (that is, <0.01%) were assigned as half of the detection limit before log-transformation. For assessment of MRD in discrete categories as in clinical practice, day 15 MRD was categorized into 4 discrete groups: <0.01%; ≥0.01 to <1%; ≥1 to <5%; and ≥5%. Day 42 MRD was categorized into 3 discrete groups: <0.01%; ≥0.01 to <1%; and ≥1%^4,37,38^.

Raw drug LC_50_ values were log-transformed and then normalized into comparable ranges based on the minimum and maximum concentrations tested for each drug, where normalized LC_50_ values fell into a range between 0 (most sensitive) and 1 (most resistant), that is, LC_50__normalized_ = (LC_5__0__measured_ − LC_50__min_)/(LC_50__max_ − LC_50__min_). To take into account the different concentration ranges tested for each drug, an alternative normalization method was also performed where the fold change from the median LC_50_ was calculated and then log-transformed, that is, log_2_(LC_50__measured_/LC_50__median_).

The association between drug LC_50_ and clinical presenting features were assessed using Mann–Whitney *U*-test or Kruskal–Wallis test. Correlations of single-drug LC_50_ with day 15 or day 42 MRD were assessed using multiple linear regression adjusting for protocol, age and WBC at diagnosis. For each patient, MRD was measured during induction therapy at day 15 and day 42 from diagnosis (day 15 and day 42 MRD). In this study, we evaluated the coefficient of linear regression (*β*), where each unit change in coefficient represents a unit change of MRD (log_10_-transformed) where a coefficient of 1.0 represents a tenfold increase of MRD, that is, a coefficient of 0.30 (or log_10_(2)) indicates that the MRD is twice as high in resistant patients compared to sensitive patients and a coefficient of −0.30 (or log_10_(0.5)) indicates that the MRD is half as high. A coefficient of zero indicates that there is no difference in MRD between resistant and sensitive patients. The Kruskal–Wallis test was used to compare the distribution of LC_50_ values between subtypes.

**Extended Data Fig. 7:**
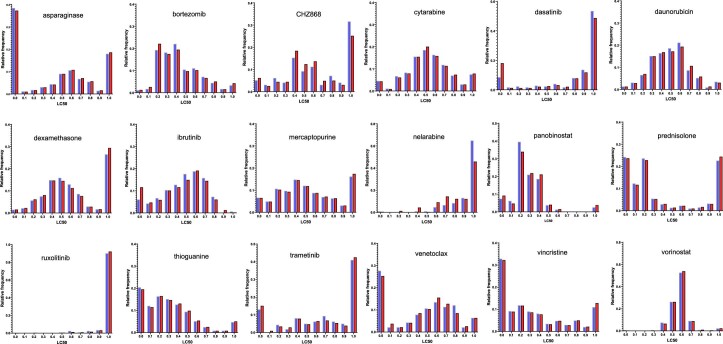
Distribution of measured vs. imputed drug LC50s. Histogram plots of LC50 distributions of measured (blue) vs imputed (red) values are shown for each drug. There are no statistically significant differences between distribution of both datasets (2-sided Chi-square test with Benjamini-Hochberg correction). Source data

Because not all patients were tested for all drugs, we also implemented LC_50_ imputation to evaluate the LC_50_ correlation across drugs. We imputed missing LC_50_ values via multivariate imputation by chained equations^55^. Overall, we constructed a total of ten imputation datasets. We recognize the limitations of multiple imputation as a statistical tool and hence also performed several analyses to confirm the validity of our imputation. First, comparing the LC_50_ distribution of measured versus imputed values, we found no statistically significant difference between the two datasets for all 18 drugs tested (chi-squared test with Benjamini–Hochberg correction) (Extended Data Fig. 7). Second, we performed leave-one-out analysis to assess imputation validity. For each measured LC_50_, we masked the value as ‘NA’ and performed the same multiple imputation procedure for 10 rounds. From these 10 datasets, we recorded the average imputed LC_50_ and the s.d. across all 10 imputations. In this analysis, 94% of observed/measured LC_50_ values fell within the 95% CI of imputed values (10 estimates), suggesting that the imputation was reasonable relative to the actual measured values and accurately reflected the variability of LC_50_. Third, we compared the linear regression coefficients of the association between day 15 MRD and measured versus imputed LC_50_ values for all 18 drugs, where the regression coefficients derived from imputed versus measured values are highly similar (Pearson’s *r* = 0.9, *P* < 0.0001).

We generated hierarchical clustering of patients based on stacking all imputed LC_50_ values across the ten imputed datasets using Manhattan distance measure. Thus, each patient had ten corresponding imputed data points, each from an imputed dataset, and received a cluster assignment for each round of imputation. A patient was assigned to a final cluster if the patient’s imputed data points were assigned to the same cluster at least five times. Hierarchical clustering of drugs was based on a correlation distance measure.

Survival outcomes were examined as EFS. EFS was calculated as the interval of time from the date of diagnosis until the date of first treatment failure (including induction failure, relapse, second malignancy and death resulting from any cause). For those who did not experience events, EFS was the time to last contact. Five-year survival probabilities and corresponding s.e. were calculated separately for each of the six drug sensitivity clusters or two dasatinib LC_50_ groups using Kaplan–Meier curves. We evaluated associations between drug sensitivity clusters/groups and EFS using the Mantel’s log-rank test^56^. Multivariable analysis of EFS was performed with the Cox proportional-hazards regression model^57^. MRD positivity (that is, ≥0.01%) was included in the multivariable analysis together with age at diagnosis, WBC count at diagnosis, leukemia subtype (B cell ALL versus T cell ALL), drug sensitivity cluster and/or dasatinib sensitivity, as relevant. All *P* values in the outcome analyses were adjusted by treatment arm (that is, TXV low risk, TXV standard/high risk, TXVI low risk, TXVI standard/high risk).

All analyses were performed with R v.3.6.3 or Prism 9 (GraphPad Software). All statistical tests were two-sided and *P* values were considered nominally significant if <0.05. Nominal *P* values are reported along with *P* values after Benjamini–Hochberg correction as appropriate.

### Role of the funding source

The funding agencies were not directly involved in the design of the study, gathering, analysis and interpretation of the data, writing of the manuscript or decision to submit the manuscript for publication.

### Reporting summary

Further information on research design is available in the Nature Portfolio Reporting Summary linked to this article.

## Online content

Any methods, additional references, Nature Portfolio reporting summaries, source data, extended data, supplementary information, acknowledgements, peer review information; details of author contributions and competing interests; and statements of data and code availability are available at 10.1038/s41591-022-02112-7.

## Supplementary information

Supplementary Information Supplementary Fig. 1 and Tables 3–9.

Reporting Summary

Supplementary Table 1 Supplementary Tables 1 and 2 – LC_50_ dataset with clinical data and RNA-seq data location

## Data Availability

Supplementary Table 1 includes all measured drug sensitivity values (LC_50_) and corresponding clinical data. RNA-seq data have been deposited in the European Genome-phenome Archive under accession nos. EGAS00001001952, EGAS00001001923, EGAS00001000447, EGAS00001000654, EGAS00001003266, EGAS00001004739, EGAS00001005084 and EGAS00001006336. Data are also available at St. Jude Cloud Genomics Platform for the Pan-Acute Lymphoblastic Leukemia dataset (https://platform.stjude.cloud/data/cohorts?dataset_accession=SJC-DS-1009), for the Real-time Clinical Genomics dataset (https://platform.stjude.cloud/data/cohorts?dataset_accession=SJC-DS-1007) and for the FPKM matrix at https://permalinks.stjude.cloud/permalinks/all-pharmacotype. Raw sequencing data are available under controlled access to ensure appropriate data usage; approval can be obtained by contacting the PCGP Steering Committee (PCGP_data_request@stjude.org). Corresponding data accessions and locations for each case are listed in Supplementary Table 2. The 1000 Genomes reference population dataset is available at https://www.internationalgenome.org/data-portal/. Source data are provided with this paper.

## Source data

Source Data Fig. 2 Source data—numbers.
Source Data Fig. 3 Source data—numbers.
Source Data Fig. 4 Source data—numbers.
Source Data Extended Data Fig. 1 Source data —numbers.
Source Data Extended Data Fig. 2 Source data—numbers.
Source Data Extended Data Fig. 3 Source data—numbers.
Source Data Extended Data Fig. 4 Source data—numbers.
Source Data Extended Data Fig. 5 Source data—numbers.
Source Data Extended Data Fig. 6 Source data—numbers.
Source Data Extended Data Fig. 7 Source data—numbers.

## Code availability

This study did not involve the development of custom code. The codes used to run specific analysis are available on GitHub at https://github.com/jjyanglab/pharmacotyping_2022.
