# Supplementary material for: Pharmacotypes across the genomic landscape of pediatric acute lymphoblastic leukemia and impact on treatment response
Source: Nat Med. 2023 Jan 5;29(1):170–9. doi: 10.1038/s41591-022-02112-7 (PMC9873558; doi:10.1038/s41591-022-02112-7)
Supplement: Supplementary file 1 — Supplementary Fig. 1 and Tables 3–9. [file 41591_2022_2112_MOESM1_ESM.pdf]

nature medicine

Article

https://doi.org/10.1038/s41591-022-02112-7

Pharmacotypes across the genomic landscape of pediatric acute lymphoblastic leukemia and impact on treatment response

In the format provided by the authors and unedited

Supplementary Information

Supplementary Figure 1

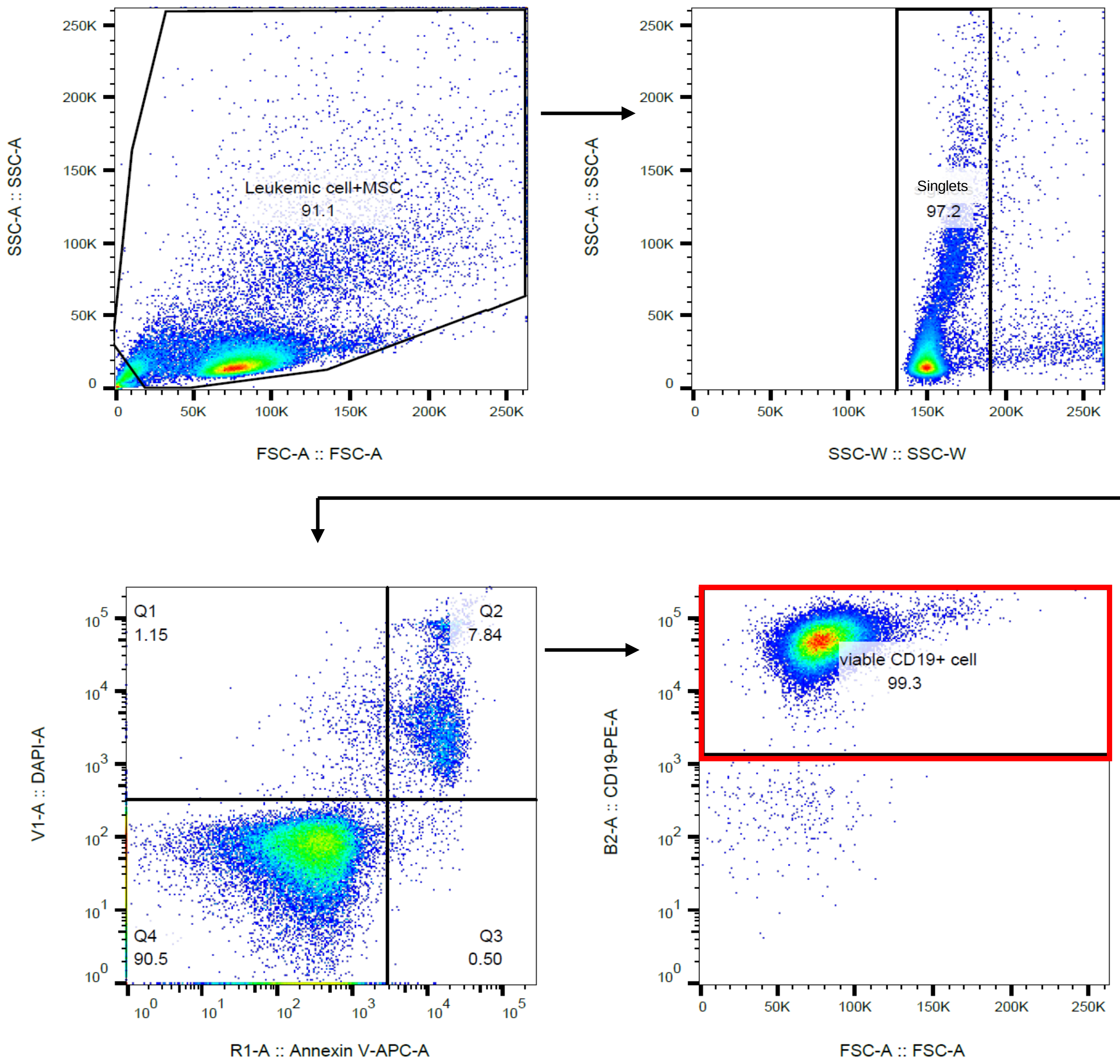

Supp Figure 1. Gating strategy for MSC-flow assay. A representative patient sample is shown. Flow data is analyzed on Flowjo. Debris was first excluded by SSC-A vs FSC-A, then singlets were gated by SSC-A vs SSC-W. Next, GFP negative cells were gated by GFP vs CD19-PE. Viable cells (annexin V and DAPI negative) were then gated by DAPI vs Annexin V-APC, and finally, viable leukemia cells (CD7 for T-ALL and CD19 for B-ALL) were further gated by CD19 or CD7 vs FSC-A.

Supplementary Tables 3-9

Supp Table 3. LC50 of each drug across clinical features

Normalized median LC50	Age (years)[#]			Sex			WBC at diagnosis (x 10^9/L)			NCI risk group			Population and ancestry				
	1 to <10	≥10	P value	F	M	P value	<50	≥50	P value	SR	HR	P value	European	African	Admixed American	Other	P value
prednisolone	**0.18**	**0.26**	**0.003***	0.17	0.21	0.013	0.18	0.19	0.880	0.18	0.20	0.055	0.19	0.17	0.17	0.19	0.223
dexamethasone	0.55	0.61	0.038	0.54	0.60	0.056	0.58	0.55	0.630	0.56	0.59	0.246	0.60	0.52	0.49	0.68	0.055
vincristine	0.23	0.16	0.066	0.20	0.24	0.512	0.21	0.24	0.416	0.23	0.20	0.579	0.22	0.25	0.08	0.14	0.031
daunorubicin	0.49	0.54	0.238	0.5	0.50	0.814	0.51	0.49	0.901	0.50	0.50	0.482	0.50	0.45	0.53	0.50	0.612
asparaginase	**0.40**	**0.62**	**0.001***	0.42	0.51	0.174	0.45	0.52	0.126	**0.37**	**0.56**	**2.6E-04***	0.47	0.47	0.50	0.39	0.773
mercaptopurine	0.48	0.49	0.840	0.48	0.49	0.651	**0.5**	**0.42**	**0.004***	0.50	0.45	0.091	0.50	0.45	0.45	0.40	0.383
thioguanine	0.26	0.25	0.896	0.24	0.27	0.224	0.27	0.22	0.092	0.27	0.23	0.083	0.29	0.17	0.19	0.21	0.006
cytarabine	0.52	0.59	0.048	0.5	0.54	0.513	0.54	0.48	0.076	0.53	0.52	0.271	0.54	0.49	0.53	0.47	0.293
nelarabine	1.0	0.98	0.383	1.0	1.0	0.954	1.0	0.98	0.120	1.0	0.98	0.052	1.0	1.0	1.0	0.87	0.084
vorinostat	0.58	0.58	0.983	0.57	0.59	0.260	0.59	0.57	0.865	0.59	0.57	0.774	0.58	0.60	0.55	0.56	0.546
panobinostat	0.23	0.28	0.511	0.24	0.23	0.440	0.24	0.21	0.062	0.24	0.21	0.596	0.24	0.28	0.21	0.2	0.285
bortezomib	0.38	0.41	0.800	0.38	0.40	0.658	0.39	0.43	0.151	0.38	0.41	0.625	0.38	0.36	0.42	0.38	0.680
CHZ868	0.6	0.55	0.738	0.61	0.56	0.749	0.67	0.51	0.626	0.67	0.55	0.945	0.77	0.41	0.51	0.49	0.332
ruxolitinib	1.0	1.0	0.888	1.0	1.0	0.591	1.0	1.0	0.935	1.0	1.0	0.481	1.0	1.0	1.0	1.0	0.935
dasatinib	0.96	0.98	0.865	1.0	0.95	0.380	**1.0**	**0.86**	**3.2E-04***	0.99	0.95	0.107	1.0	0.88	0.92	0.92	0.191
ibrutinib	0.52	0.50	0.664	0.53	0.50	0.635	0.51	0.53	0.997	0.51	0.53	0.280	0.54	0.51	0.42	0.45	0.779
trametinib	0.79	0.75	0.935	0.79	0.75	0.512	0.78	0.78	0.657	0.76	0.82	0.270	0.78	0.98	0.65	0.69	0.411
venetoclax	0.53	0.46	0.847	0.52	0.51	0.753	0.47	0.57	0.017	0.46	0.57	0.011	0.53	0.56	0.45	0.45	0.776

[#]7 patients aged less than 1 year of age not included due to small numbers of measurements

Nominal P values determined by 2-sided Mann-Whitney test or Kruskal-Wallis test are shown. Those with P<0.05 after adjustment by Benjamini-Hochberg procedure are marked in bold and by an asterix (*) and are as follows: prednisolone and age, p-adjust =0.027; asparaginase and age, p-adjust=0.018; mercaptopurine and WBC, p-adjust=0.036; dasatinib and WBC, p-adjust=0.006; asparaginase and NCI, risk, p-adjust=0.005.

Abbreviations: WBC, white blood cell count; NCI, National Cancer Institute; SR, standard risk; HR, high risk

Supp Table 4. Number of tested samples in each drug-subtype combination

Drug	*ETV6-RUNX1*	*ETV6-RUNX1*-like	Hyperdiploid	*DUX4*	*PAX5*alt	*TCF3-PBX1*	*ZNF384*	T-ALL	ETP	*BCR-ABL1*	*BCR-ABL1*-like	*CRLF2*	*KMT2A*	iAMP21	*MEF2D*	Near haploid	B-other
asparaginase	151	11	132	29	25	26	6	89	14	17	12	14	27	6	6	5	40
bortezomib	48	2	42	11	8	5	1	21	4	5	4	5	6	4	1	1	12
CHZ868	27	1	19	5	2	2	1	8		5	2	4	5	2	1	1	13
cytarabine	90	9	76	18	15	15	5	57	6	9	4	7	20	2	3	2	28
dasatinib	42	2	37	12	8	3	1	23	3	6	3	9	6	3	2	1	14
daunorubicin	46	2	39	9	7	5	1	72	11	5	4	5	6	4	1	1	12
dexamethasone	118	10	105	22	24	23	5	54	6	11	6	11	21	4	5	6	36
ibrutinib	40	2	36	11	7	3	1	20	2	6	2	9	5	3	1	1	14
mercaptopurine	170	13	146	28	28	25	4	79	12	17	12	13	22	8	6	8	47
nelarabine	34		22	6	4	3		11	4	3	3	2	3	2	1		8
panobinostat	16	1	20	6	5	2		15		2	1	2	4	2	1	1	2
prednisolone	156	11	136	29	28	25	6	82	15	15	9	14	24	7	8	7	43
ruxolitinib	28	2	32	8	7	1	1	13	1	4	2	5	4	3	2	1	7
thioguanine	123	11	110	21	23	20	6	58	8	13	7	10	18	5	5	6	36
trametinib	40	2	37	10	7	3	1	18	2	6	2	8	5	3	1	1	14
venetoclax	37	1	30	8	4	2	1	16	2	6	2	7	5	3	1	1	14
vincristine	157	10	130	25	24	23	5	91	14	15	7	14	23	7	6	5	42
vorinostat	40		25	8	4	3	1	13	4	5	3	5	5	3	1	1	10

Supp Table 5. Nominal and adjusted P-values for comparisons of drug sensitivity between leukemia subtypes

Drug	Comparisons	2-sided nominal P value	P value after Benjamini-Hochberg correction (p-adjust)
Prednisolone, vincristine, daunorubicin, asparaginase (PVDL)	*ETV6-RUNX1* vs. remainder	1.90E-18	3.70E-17
	Hyperdiploid vs. remainder	0.004	0.012
	BCR-ABL1 vs *ETV6-RUNX1*	0.001	0.010
	BCR-ABL1-like vs *ETV6-RUNX1*	5.27E-07	5.90E-05
	KMT2A vs *ETV6-RUNX1*	7.76E-09	9.60E-07
	DUX4 vs *ETV6-RUNX1*	0.0006	0.008
asparaginase	*DUX4* vs *ETV6-RUNX1*	1.60E-11	1.10E-08
	ETV6-RUNX1 vs. *ETV6-RUNX1*-like	0.013	0.110
cytarabine	*NUTM1* vs. remainder	0.007	0.047
	T-ALL vs ETP-ALL	0.020	0.147
dasatinib	T-ALL vs remainder	1.75E-07	2.60E-06
	TCF3-PBX1 vs remainder	0.013	0.049
	BCR-ABL1 vs remainder	3.05E-07	2.18E-06
daunorubicin	T-ALL vs ETP-ALL	0.001	0.128
ibrutinib	*BCR-ABL1* vs remainder	0.002	0.034
mercaptopurine	*NUTM1* vs. remainder	0.002	0.010
	T-ALL vs ETP-ALL	0.017	0.080
prednisolone	T-ALL vs ETP-ALL	0.009	0.045
thioguanine	*NUTM1* vs. remainder	0.0003	0.003
	T-ALL vs ETP-ALL	0.006	0.043
trametinib	*ETV6-RUNX1* vs. *ETV6-RUNX1*-like	0.002	0.283
venetoclax	Hyperdiploid vs. remainder	4.08E-05	5.33E-04
vincristine	T-ALL vs ETP-ALL	0.0005	0.006

For subtype A vs subtype B comparisons, correction for multiple testing performed for number of pairwise comparisons made (up to N=253).
For subtype A vs the remainder comparisons, correction for multiple testing performed for number of subtypes compared (up to N=23).

Supp Table 6. Comparison of included vs. non-included patients from TXV/TXVI cohorts

Clinical characteristics		Not Included (N=462)		Included (N=634)		
		N	%	N	%	P value*
Subtype	T-ALL	72	15.6%	108	17.0%	0.522
	B-ALL	390	84.4%	526	83.0%	
Treated risk group	LR	221	48.9%	274	44.0%	0.11
	SR/HR	231	51.1%	349	56.0%	
Age group (years)	<1	5	1.1%	7	1.1%	0.123
	1 to <10	320	74.8%	474	69.3%	
	≥10	137	24.1%	153	29.7%	
WBC at diagnosis ($x10^9$/L)	<50	375	71.9%	456	81.2%	0.0004
	≥50	87	28.1%	178	18.8%	
D42 MRD	MRD negative (<0.01%)	370	83.7%	521	83.5%	0.925
	MRD positive (≥0.01%)	72	16.3%	103	16.5%	

*Nominal P values as shown and determined by 2-sided Chi-square test

Abbreviations: WBC, white blood cell count; SR, standard risk; HR, high risk

Supp Table 7. Multivariable analysis of drug sensitivity clusters with event-free-survival in whole cohort

Prognostic factor		HR	95% CI	P value*
Drug sensitivity cluster	Cluster I	5.31	1.35-20.84	0.025
	Cluster II	2.57	0.8-8.2	
	Cluster III (ref)	1.0	-	
	Cluster IV	0.79	0.34-1.84	
	Cluster V	1.11	0.3-4.09	
	Cluster VI	1.98	0.97-4.07	
Minimal residual disease	Negative (<0.01%) (ref)	1.0	-	0.015
	Positive (≥0.01%)	1.99	1.14-3.47	
WBC at diagnosis ($x10^9/L$)	<50 (ref)	1.0	-	0.029
	≥50	1.88	1.07-3.28	
Age at diagnosis (years)	<1	2.40	0.55-10.47	0.477
	1 to 10 (ref)	1.0	-	
	≥10	1.23	0.71-2.13	
B vs T subtype	B-ALL (ref)	1.0	-	0.227
	T-ALL	0.66	0.33-1.31	

*P values determined by 2-sided Cox proportional hazard's regression test. P values are adjusted for treatment arm i.e. TXV low risk, TXV standard/high risk, TXVI low risk, TXVI standard/high risk

Supp Table 8. Multivariable analysis of dasatinib sensitivity with event-free survival in T-ALL

	Prognostic factor	HR	95% CI	P value
Dasatinib sensitivity	LC50 <0.25	3.23	1.03-10.14	0.044
	LC50 ≥0.25 (ref)	1.0	-	
Minimal residual disease	Negative (<0.01%) (ref)	1.0	-	
	Positive (≥0.01%)	1.65	0.52-5.27	0.398
Age at diagnosis (years)	1 to 10 (ref)	1.0	-	
	≥10	1.06	0.38-2.90	0.905
WBC at diagnosis ($x10^9/L$)	<50 (ref)	1.0	-	
	≥50	1.51	0.44-5.15	0.511

*P values determined by 2-sided Cox proportional hazard's regression test. P values are adjusted for treatment arm i.e. TXV standard/high risk, TXVI standard/high risk

Supp Table 9. Tested concentrations for 18 drugs in ex vivo pharmacotyping assays

Levels	Drug concentrations (units)																	
	Asparaginase (IU/ml)	Bortezomib (nM)	CHZ868 (nM)	Cytarabine (µM)	Dasatinib (nM)	Daunorubicin (µM)	Dexamethasone (µM)	Ibrutinib (µM)	Mercaptopurine (µM)	Nelarabine (µM)	Panobinostat (nM)	Prednisolone (µM)	Ruxolitinib (nM)	Thioguanine (µM)	Trametinib (nM)	Venetoclax (nM)	Vincristine (µM)	Vorinostat (nM)
1	0.0032	0.98	0.1	0.04	0.1	0.004	0.00035	1.5625	91.8	1.03	0.98	0.015	0.1	9.35	0.01	0.001	0.0017	102.88
2	0.016	3.9	1	0.16	1	0.014	0.0028	3.125	183.6	3.09	3.9	0.125	1	18.69	0.1	0.01	0.0132	308.64
3	0.08	15.6	10	0.64	10	0.055	0.023	6.25	367.25	9.26	15.9	0.98	10	37.38	1	0.1	0.1058	925.93
4	0.4	62.5	100	2.56	100	0.22	0.181	12.5	734.5	27.8	62.5	7.87	100	74.75	10	1	0.8464	2777.78
5	2	250	1000	10.27	1000	0.89	1.45	25	1469	83.3	250	62.9	1000	149.5	100	10	6.7711	8333.33
6	10	1000	10000	41.1	10000	3.55	11.6	50	2938	250	1000	503.5	10000	299	1000	100	54.169	25000
