# Supplementary material for: Pharmacotypes across the genomic landscape of pediatric acute lymphoblastic leukemia and impact on treatment response
Source: Nat Med. 2023 Jan 5;29(1):170–9. doi: 10.1038/s41591-022-02112-7 (PMC9873558; doi:10.1038/s41591-022-02112-7)
Supplement: Supplementary file 2 — Reporting Summary [file 41591_2022_2112_MOESM2_ESM.pdf]

nature research

Corresponding author(s): Jun J. Yang

Last updated by author(s): Oct 15, 2022

Reporting Summary

Nature Research wishes to improve the reproducibility of the work that we publish. This form provides structure for consistency and transparency in reporting. For further information on Nature Research policies, see our Editorial Policies and the Editorial Policy Checklist.

Statistics

For all statistical analyses, confirm that the following items are present in the figure legend, table legend, main text, or Methods section.

n/a	Confirmed	
☐	☒	The exact sample size (*n*) for each experimental group/condition, given as a discrete number and unit of measurement
☐	☒	A statement on whether measurements were taken from distinct samples or whether the same sample was measured repeatedly
☐	☒	The statistical test(s) used AND whether they are one- or two-sided<br>*Only common tests should be described solely by name; describe more complex techniques in the Methods section.*
☐	☒	A description of all covariates tested
☐	☒	A description of any assumptions or corrections, such as tests of normality and adjustment for multiple comparisons
☐	☒	A full description of the statistical parameters including central tendency (e.g. means) or other basic estimates (e.g. regression coefficient) AND variation (e.g. standard deviation) or associated estimates of uncertainty (e.g. confidence intervals)
☐	☒	For null hypothesis testing, the test statistic (e.g. *F*, *t*, *r*) with confidence intervals, effect sizes, degrees of freedom and *P* value noted<br>*Give P values as exact values whenever suitable.*
☒	☐	For Bayesian analysis, information on the choice of priors and Markov chain Monte Carlo settings
☒	☐	For hierarchical and complex designs, identification of the appropriate level for tests and full reporting of outcomes
☐	☒	Estimates of effect sizes (e.g. Cohen's *d*, Pearson's *r*), indicating how they were calculated

Our web collection on statistics for biologists contains articles on many of the points above.

Software and code

Policy information about availability of computer code

Data collection	See below for description of collection (and analyses, full description of data collection and analyses can be found in methods and supplementary methods). Flow cytometry (FACS) - BO FACS Aria lllu machine & BO FACS LSR II machine (BD FACS Diva Software V8.0); MACS QuantX (FlowJo software Vl0.5.3/10.6.1); Sequencing (RNA-seq) - HiSeq 2000/2500 or NovaSeq 6000 platform; MTT (measurement of MTT absorbance) - BioTek Synergy H4.
Data analysis	STAR (version 2.4.2a)<br>CICERO (in-house version)<br>FusionCatcher (version 1.0)<br>Integrative Genomics Viewer 2.10<br>RSEM v1 .2.2887<br>HTSeq (version 0.6.0)<br>BD FACS Diva Software (9.0)<br>R programming language (version 4.0.3) and RStudio (2022.02.0+443)<br>DESeq2 R package (version 1.18.1, RNA-seq gene expression analysis)<br>sva R package (version 3.26.0, batch effect correction)<br>Rtsne R package (version 0.13, tSNE analysis)<br>Samtools (version 1.3.1)<br>iAdmix (https://github.com/eliorav/iAdmix, Lbfgfb.3.0)<br>Picard (version 1.129)<br>GATK (version 3.7)<br>Pamr R package (versoin 1.55, PAM prediction)<br>Genome Studio (Illumina, version 2.0.3)<br>Prism (GraphPad, version 7.0)<br>MICE R package (version 3.13.0)

ClustVis 1.0 (biit.cs.ut.ee/clustvis)
Survival R package (version 3.3-1)
glmnet R package (version 4.1.1)
corrplot R package (version 0.92)

This study did not involve the development of custom code. The codes used to run specific analysis are available on Github at https://github.com/jjyanglab/pharmacotyping_2022.

For manuscripts utilizing custom algorithms or software that are central to the research but not yet described in published literature, software must be made available to editors and reviewers. We strongly encourage code deposition in a community repository (e.g. GitHub). See the Nature Research guidelines for submitting code & software for further information.

Data

Policy information about availability of data

All manuscripts must include a data availability statement. This statement should provide the following information, where applicable:

- Accession codes, unique identifiers, or web links for publicly available datasets
- A list of figures that have associated raw data
- A description of any restrictions on data availability

Supplementary Table 1 includes all measured drug sensitivity values (LC50) and corresponding clinical data.
RNAseq data have been deposited in the European Genome-phenome Archive: EGAS00001001952, EGAS00001001923, EGAS00001000447, EGAS00001000654, EGAS00001003266, EGAS00001004739, EGAS00001005084, and EGAS00001006336. Data are also available at St. Jude Cloud for the Pan-Acute Lymphoblastic Leukemia (PanALL) dataset: https://platform.stjude.cloud/data/cohorts?dataset_accession=SJC-DS-1009, for the Real-time Clinical Genomics dataset: https://platform.stjude.cloud/data/cohorts?dataset_accession=SJC-DS-1007, and for the FPKM matrix at https://permalinks.stjude.cloud/permalinks/all-pharmacotype.
Raw sequencing data are available under controlled access to ensure appropriate data usage, and approval can be obtained by contacting the PCGP Steering Committee (PCGP_data_request@stjude.org). Corresponding data accessions and locations for each case are listed in Supplementary Table 2.
The 1000Genomes reference population dataset is available at https://www.internationalgenome.org/data-portal/.
Figures that have associated raw data: Figures 2,3,4,5; Ext Data Figures 1,2,3,4,5,6,7

Field-specific reporting

Please select the one below that is the best fit for your research. If you are not sure, read the appropriate sections before making your selection.

☒ Life sciences ☐ Behavioural & social sciences ☐ Ecological, evolutionary & environmental sciences

For a reference copy of the document with all sections, see nature.com/documents/nr-reporting-summary-flat.pdf

Life sciences study design

All studies must disclose on these points even when the disclosure is negative.

Sample size	No sample sized calculation was performed. Pharmacotyping was performed based on availability of diagnostic bone marrow for patients enrolled on St Jude Total therapy trials. It should be noted that the number of ALL cases included in the current study is much greater than prior publications on ALL pharmacotyping.
Data exclusions	1. RNA-seq data with low sequencing coverage (30-fold coverage <15%) were excluded since insufficient coverage and depth will lead to unreliable and biased evaluation of gene expression level. We have tested and established this criteria in our previous studies (Gu et al., Nat Commun, 2016; Alexander et al., Nature, 2018).<br>2. Samples were subject to pharmacotyping only if all of the following pre-established criteria were met after Ficoll/magnetic bead enrichment: Viability > 80%, Blast > 85%. Otherwise, these samples were excluded from drug sensitivity testing. After pharmamacotyping was performed, no downstream data was excluded from analysis.
Replication	Pharmacotyping was performed all successfully in duplicates.<br>No replication of genomic analysis was performed, as this is not required when performing high-depth and high-coverage next generation sequencing (RNAseq).
Randomization	Nothing to disclose. This is not an intervention study, so randomization is not applicable.
Blinding	Nothing to disclose. This is not an intervention study, so blinding is not applicable.

Reporting for specific materials, systems and methods

We require information from authors about some types of materials, experimental systems and methods used in many studies. Here, indicate whether each material, system or method listed is relevant to your study. If you are not sure if a list item applies to your research, read the appropriate section before selecting a response.

Materials & experimental systems

n/a	Involved in the study
☐	☒ Antibodies
☒	☐ Eukaryotic cell lines
☒	☐ Palaeontology and archaeology
☒	☐ Animals and other organisms
☐	☒ Human research participants
☐	☒ Clinical data
☒	☐ Dual use research of concern

Methods

n/a	Involved in the study
☒	☐ ChIP-seq
☐	☒ Flow cytometry
☒	☐ MRI-based neuroimaging

Antibodies

Antibodies used

For flow cytometry for MSC-flow assay, the following antibodies were used:
Biolegend: Human CD7-PE (clone 4H9, cat #395604), Human CD19-PE (clone SJ25C1, cat #363004)
BD Biosciences: Annexin-V APC (AB_2868885, cat #550475)

Human CD19-PE: https://www.biolegend.com/en-us/products/pe-anti-human-cd19-antibody-10263
Human CD7-PE: https://www.biolegend.com/en-us/products/pe-anti-human-cd7-antibody-18586
Annexin-V APC: https://www.bdbiosciences.com/en-us/products/reagents/flow-cytometry-reagents/research-reagents/single-color-antibodies-ruo/apc-annexin-v.550475

Validation

All antibodies for flow cytometry were validated for detecting human proteins by the manufacturer and confirmed for each specific application using cells of known origin and differentiation state and compared to isotype controls and cells that are known to express or lack the antigen. Antibodies used for flow cytometry analysis were validated by the SJCRH Flow Core facility.

Validation information for each product can be found on the company websites with the links listed above. For example, for Human CD19-PE, the following statement is on the manufacturer's technical data sheet: "Each lot of this antibody is quality control tested by immunofluorescent staining with flow cytometric analysis. For flow cytometric staining, the suggested use of this reagent is 5 µl per million cells in 100 µl staining volume or 5 µl per 100 µl of whole blood." Details can be found at: https://www.biolegend.com/en-us/products/pe-anti-human-cd19-antibody-10263?pdf=true&displayInline=true&leftRightMargin=15&topBottomMargin=15&filename=PE%20anti-human%20CD19%20Antibody.pdf&v=20220330063155

Human research participants

Policy information about studies involving human research participants

Population characteristics

All human subjects were treated for newly diagnosed ALL on St Jude Total Therapy XV, XVI or XVII clinical trials; no subjects were enrolled solely for the pharmacotyping study.

In this study, we collected banked tumor samples for RNA-sequencing as well as pharmacotyping, and correlated with clinical data generated from their treatment on the above trials.

In the Total Therapy trials, sex was self reported. Sex was not considered as an inclusion/exclusion criterion of the original trials. Sex was also not considered in the design of the pharmacotyping study.

Covariates relevant to this study included: age at diagnosis, white blood cell count (WBC) count at diagnosis, RNA-seq derived molecular subtype, genetically-defined ancestry, and treatment arm.

Recruitment

Patients were recruited to Total Therapy Trials via our referral network system. Patients were enrolled on these trials and study after providing consent and assent as appropriate. Recruitment for participation was performed in the clinical setting in the hospital clinics/wards, at diagnosis or initiation of treatment for their leukemia.
No patients were specifically recruited for the pharmacotyping study. That said, there are potential biases in sample representation that may influence the results: 1. this ALL cohort reflects patient population at a single center (St. Jude) and therefore may not represent the full diversity of pediatric ALL (especially ancestry); and 2. Not all patients treated on Total Therapy trials had sufficient materials for pharmacotyping. We compared patient characteristics between those included vs not included in the pharmacotyping study and these two groups are highly similar (Supp Table 6).

Ethics oversight

Institutional Review Board at St Jude Children's Research Hospital

Note that full information on the approval of the study protocol must also be provided in the manuscript.

Clinical data

Policy information about clinical studies
All manuscripts should comply with the ICMJE guidelines for publication of clinical research and a completed CONSORT checklist must be included with all submissions.

Clinical trial registration

Clinicaltrials.gov NCT00137111, NCT00549848, NCT03117751

Study protocol	Study protocol can be obtained on Clinicaltrials.gov
Data collection	Total Therapy studies were conducted from 2000 to 2021: Total XV stopped in 2010, Total XVI stopped in 2020, with Total XVII currently still ongoing. Pharmacotyping data collection occurred along with these trials as the patients were enrolled and samples were processed for drug sensitivity testing.
Outcomes	Survival outcomes of the Total Therapy trials were pre-defined. We examined event-free survival (EFS) in our study. EFS was calculated as the interval of time from the date of diagnosis until the date of first treatment failure (including induction failure, relapse, second malignancy, and death resulting from any cause). For those who did not experience events, EFS was the time to last contact. 5-year survival probabilities and corresponding standard errors (SE) were calculated separately for each of the six drug sensitivity clusters or two dasatinib LC50 groups using Kaplan-Meier curves. We evaluated associations between drug sensitivity clusters/groups and EFS using the Mantel's log-rank test. Multivariable analysis of EFS were performed with the Cox proportional hazards regression model15. Minimal residual disease (MRD) positivity (i.e. ≥0.01%) was included in the multivariable analysis together with drug sensitivity cluster or dasatinib sensitivity. All P-values in outcomes analyses were adjusted by treatment arm (i.e. TXV low risk, TXV standard/high risk, TXVI low risk, TXVI standard/high risk).

Flow Cytometry

Plots

Confirm that:

☒ The axis labels state the marker and fluorochrome used (e.g. CD4-FITC).

☒ The axis scales are clearly visible. Include numbers along axes only for bottom left plot of group (a 'group' is an analysis of identical markers).

☒ All plots are contour plots with outliers or pseudocolor plots.

☒ A numerical value for number of cells or percentage (with statistics) is provided.

Methodology

Sample preparation	The description of sample preparation and MSC-flow assay can be found in the Methods section. For FACS sorting and flow analyses of leukemia cells, cells were resuspended in FACS buffer (PBS supplemented with 2% Fetal Calf Serum and 0.25mM EDTA), they were then stained with proper antibodies, DAPI and/or annexin V, and washed twice with FACS buffer before being subjected to FACS sorting.
Instrument	BD FACS Aria IIIu machine, BD FACS LSR II machine, MACS QuantX.
Software	BD FACS Diva Software, Flowjo
Cell population abundance	Cell population abundance under drug treatment was compared to cell population abundance treated with vehicle alone to derive<br>It is also to be noted that flow cytometry was used in the patient treatment and pharmacotyping process to determine MRD/drug LC50; however there are no flow cytometry plots in our analyses or results for this paper.
Gating strategy	Debris was first excluded by SSC-A vs FSC-A, then singlets were gated by SSC-A vs SSC-W. Next, GFP negative cells were gated by GFP vs CD19-PE. Viable cells (annexin V and DAPI negative) were then gated by DAPI vs Annexin V-APC, and finally, viable leukemia cells (CD7 for T-ALL and CD19 for B-ALL) were further gated by CD19 or CD7 vs FSC-A.

☒ Tick this box to confirm that a figure exemplifying the gating strategy is provided in the Supplementary Information.
